# Feasibility and Acceptability of Web-Based Structured Oral Examinations for Postgraduate Certification: Mixed Methods Preliminary Evaluation

**DOI:** 10.2196/40868

**Published:** 2024-03-06

**Authors:** Vanessa Burch, Jessica McGuire, Eric Buch, Mike Sathekge, Francis M'bouaffou, Flavia Senkubuge, Johannes Fagan

**Affiliations:** 1 The Colleges of Medicine of South Africa Cape Town South Africa; 2 The University of Cape Town Cape Town South Africa; 3 University of Pretoria Pretoria South Africa

**Keywords:** web-based certification examinations, web-based structured oral examinations, medical education, specialist and subspecialist examinations, structured oral examinations, Colleges of Medicine of South Africa

## Abstract

**Background:**

The COVID-19 pandemic disrupted postgraduate certification examinations globally. The Colleges of Medicine of South Africa continued hosting certification examinations through the pandemic. This was achieved by effecting a rapid transition from in-person to web-based certification examinations.

**Objective:**

This formative evaluation explored candidates’ acceptability of web-based structured oral examinations (SOEs) hosted via Zoom (Zoom Communications Inc). We also reported the audiovisual quality and technical challenges encountered while using Zoom and candidates’ overall experience with these examinations conducted during the early part of the COVID-19 pandemic. Additionally, performance in web-based certification examinations was compared with previous in-person certification examinations.

**Methods:**

This mixed methods, single-arm evaluation anonymously gathered candidates’ perceptions of web-based SOE acceptability, audiovisual quality, and overall experience with Zoom using a web-based survey. Pass rates of web-based and previous in-person certification examinations were compared using chi-square tests, with a Yates correction. A thematic analysis approach was adopted for qualitative data.

**Results:**

Between June 2020 and June 2021, 3105 candidates registered for certification examinations, 293 (9.4%) withdrew, 2812 (90.6%) wrote, and 2799 (99.9%) passed, and 1525 (54.2%) were invited to a further web-based SOE. Examination participation was 96.2% (n=1467). During the first web-based examination cycle (2020), 542 (87.1%) of 622 web-based SOE candidates completed the web-based survey. They reported web-based SOEs as fair (374/542, 69%) and adequately testing their clinical reasoning and insight (396/542, 73.1%). Few would have preferred real patient encounters (173/542, 31.9%) or in-person oral examinations (152/542, 28%). Most found Zoom acceptable (434/542, 80%) and fair (396/542, 73.1%) for hosting web-based SOEs. SOEs resulted in financial (434/542, 80%) and time (428/542, 79%) savings for candidates. Many (336/542, 62%) supported the ongoing use of web-based certification examinations. Only 169 technical challenges in using Zoom were reported, which included connectivity-related issues, poor audio quality, and poor image quality. The thematic analysis identified 4 themes of positive and negative experiences related to web-based SOE station design and content, examination station environment, examiner-candidate interactions, and personal benefits for candidates. Our qualitative analysis identified 10 improvements for future web-based SOEs. Candidates achieved high pass rates in web-based certification examinations in 2020 (1583/1732, 91.39%) and 2021 (850/1067, 79.66%). These were significantly higher (2020: N=8635; *χ*^2^_1_=667; *P*<.001; 2021: N=7988; *χ*^2^_1_=178; *P*<.001) than the previous in-person certification examination pass rate of 58.23% (4030/6921; 2017-2019).

**Conclusions:**

Web-based SOEs conducted by the Colleges of Medicine of South Africa during the COVID-19 pandemic were well received by candidates, and few technical difficulties were encountered while using Zoom. Better performance was observed in web-based examinations than in previous in-person certification examinations. These early findings support the ongoing use of this assessment method.

## Introduction

### Background

The COVID-19 pandemic severely disrupted the postgraduate specialty and subspecialty certification examinations globally [1-5]. The Colleges of Medicine of South Africa (CMSA) continued hosting postgraduate certification examinations during the pandemic [6]. This decision ensured that the national pipeline of specialist workforce would not be disrupted and that the careers of international medical graduates completing postgraduate training in South Africa would not be unduly delayed. Hosting these examinations during the pandemic was achieved by effecting a transition from in-person to web-based written and oral examinations within a period of 8 weeks.

### Prepandemic Certification Examinations

CMSA, a nonprofit organization founded in 1954, comprises 29 constituent member colleges, each representing a primary specialty practiced in South Africa, such as College of Pediatricians, College of Surgeons, College of Physicians, College of Family Physicians, College of Obstetricians and Gynecologists, College of Anesthesiologists, and so on [6]. CMSA conducts certification examinations at the beginning (entry level) and end of postgraduate training (exit level) for all specialties and subspecialities registered with the South African statutory medical licensing authority, the Health Professions Council of South Africa [7]. Currently, there are 65 specialties (pediatrics, internal medicine, general surgery, ophthalmology, otorhinolaryngology, anesthetics, etc) and 30 subspecialties (pediatric pulmonology in the specialty of pediatrics, trauma surgery in the specialty of general surgery, gynecological oncology in the specialty of obstetrics and gynecology, etc) practiced in South Africa. For the purposes of this paper, the term, postgraduate certification examinations, denotes all postgraduate certification examinations conducted by CMSA for all specialties and subspecialities practiced in South Africa. These examinations are similar to other international postgraduate certification examinations such as *board certification* examinations offered by the American Board of Medical Specialties [8] or *membership* examinations offered by Royal Colleges in the United Kingdom, for example, the College of Anesthesiologists of Ireland [9]. Before the COVID-19 pandemic, CMSA certification included handwritten examinations, and successful candidates were invited for in-person, patient-based, clinical examinations and in-person, unstructured oral examinations (viva voce).

### Transition to Virtual Certification Examinations

The transition occurred during the first 8-week *hard lockdown phase* of the pandemic, which included closure of all public facilities except for emergency care, food, and health care; complete ban on in-person meetings and any form of intercity travel; and national curfew from 9 PM to 6 AM [10]. Weekly web-based meetings were used to provide staff with information updates and training, supplemented by digital information manuals and web-based training sessions for all examiners and candidates learning to use Zoom (Zoom Communications Inc), an interactive web-based software meeting platform.

Handwritten examinations were replaced by web-based written examinations (short-answer questions and single, best-answer questions) using commercially available software. In-person, patient-based examinations were largely discontinued, except where geographically decentralized in-person clinical examinations could be offered in COVID-19–compliant settings. For all member colleges, a new emphasis was placed on developing web-based structured oral examinations (SOEs) to assess diagnostic reasoning, clinical decision-making, and patient management, which were previously assessed during real patient encounters. It was acknowledged that web-based SOEs could not assess bedside clinical skills (history taking and physical examination), as was previously done. This compromise, accepted as an emergency measure during the pandemic, was predicated on an agreement that implementation of workplace-based assessment in South African postgraduate training would be prioritized after the pandemic [10-12].

### Design of Web-Based SOE

Web-based SOEs were designed as case scenarios, each comprising a case description with supplementary information including laboratory test results, photographs, video clips, and radiographic and histopathological images, where appropriate. The case scenarios were prepared by national panels of examiners working remotely via Zoom. Each scenario was prepared as a Microsoft PowerPoint presentation, which was *screen shared* with candidates during the oral examination hosted via Zoom. Candidates answered the standardized questions posed by examiners working alone or in pairs. Zoom calls were also attended by moderators and trainee examiners (observers). Examiners scored the candidates’ responses independently during Zoom calls using downloaded and printed memoranda, and examiners’ handwritten notes were digitally transcribed and submitted with final examination scores to conveners at the conclusion of the examination proceedings. All Zoom calls were booked and collated on timetables with embedded hyperlinks for individual calls, which were shared with the examiner panels 1 week before the examination events. Individual Zoom calls varied in length from 15 to 60 minutes, depending on the number of case scenarios discussed per call. Candidates undertaking a web-based SOE were typically examined on 4 to 12 case scenarios, depending on the specialty or subspecialty.

### Conducting Virtual Certification Examinations

CMSA set up 14 examination venues (8 in South Africa and 6 elsewhere in southern Africa) equipped with 12-inch laptop computers; 24-inch high-resolution monitors; and internet connectivity using wireless, microwave, and fiber technology. Owing to electrical power supply interruptions (load shedding) in South Africa, all examination venues were equipped with dual power supply arrangements. On-site IT support and on-site proctoring by trained staff were provided at all venues.

Candidates undertook all web-based examinations (written and oral) at an examination venue closest to their home (<3 h travel by road). Overnight accommodation was not permitted. National occupational health and safety COVID-19 protocols were observed at all examination venues [13]. Examiners joined the Zoom calls from their home or place of work, whichever was more convenient and had better bandwidth and a stable electricity supply.

### Process of Evaluation

When CMSA implemented web-based SOEs for postgraduate certification examinations in early 2020, there were few reports in the literature. There was little reference to their use for high-stakes postgraduate specialist certification processes [14-17]. Most referred to in-person SOEs for undergraduate students [18-21]. Given the paucity of data, we set up a preliminary evaluation during the initial period of implementation to obtain early insights into candidates’ perceptions about the acceptability of this assessment method and their performance in web-based examinations as compared with in-person certification examinations. Despite its known limitations, we structured our evaluation process on the Kirkpatrick model of program evaluation (reaction, learning, behavior, and results) using four questions to address level 1 (reaction) and level 4 (results) of the model [22-25]:

1. Are web-based SOEs acceptable to examination candidates?
2. What technical challenges did examination candidates encounter while using Zoom?
3. On the basis of candidates’ experiences, how could we improve the web-based SOE experience?
4. Are pass rates for web-based and in-person certification examinations different?

## Methods

### Design

The evaluation was conducted as a cross-sectional observational study using a mixed methods design.

### Participants

The study population included all postgraduate certification examination candidates who were invited to undertake web-based SOEs after successfully completing the written component of the national certification examinations in 2020 and 2021.

### Procedure

The first cohort of examination candidates who undertook a web-based SOE between June 1, 2020, and November 15, 2020, was invited to complete the web-based survey immediately after completing their web-based SOE at one of the examination venues. A web-based study information leaflet was provided to potential participants before recruitment. The web-based questionnaire was administered on laptop computers at the examination venues. Participants were recruited to the study before releasing any examination results.

### Survey Design

The survey, designed as a Google Form, consisted of 28 items (Multimedia Appendix 1). A total of 23 closed-ended questions focused on candidates’ perceptions of the adequacy, fairness, and quality of web-based SOEs and the technical adequacy and personal time and cost savings of using Zoom to host SOEs. Closed-ended questions required either a binary (yes or no) response or a 5-point Likert-scale response (completely disagree, disagree, neutral, agree, or completely agree). Candidates’ acceptability of web-based SOEs was defined as candidates’ perceptions of the adequacy of the web-based assessment process, overall fairness, and quality of examination material used. Parameters used to determine the adequacy of the assessment process included the following: adequate assessment of clinical reasoning, judgment, insight, and decision-making; appropriate complexity of case scenarios and questions; appropriate duration of examination; appropriate time allocation per case scenario; and preference for in-person patient encounter or in-person examiner. Fairness was explored in terms of perceived overall fairness and the use of more case scenarios than previously during in-person examinations. Quality of examination material referred to clarity of material presented during the Zoom call. Candidates’ acceptability of Zoom as the hosting platform was defined in terms of perceptions of (1) overall acceptability and fairness of the web-based assessment process, (2) audiovisual quality of Zoom, and (3) personal benefits of web-based SOEs to examination candidates (time and financial savings). The survey also included 5 open-ended questions, which required a typed text response. These questions explored candidates’ overall positive and negative experiences regarding the web-based examination process. The survey was administered immediately after the examination proceedings, and we chose to anonymize all the information so as to allay candidate concerns about possible bias when deciding whether to participate in the survey. They only reported about the examination venue attended and certification examination undertaken.

### Quantitative Data Analysis

Quantitative data from the web-based questionnaire were exported into a Microsoft Excel spreadsheet before analysis. For questions using a Likert-scale response, answers were reported in 3 categories: agree (strongly agree and agree responses), neutral (neutral responses), and disagree (strongly disagree and disagree responses). Percentages were calculated and rounded to 1 decimal point.

The respective pass rates for the first 2 cycles of web-based certification examinations, 2020 and 2021, were compared with the overall pass rate for the preceding 6 cycles of in-person certification examinations conducted between 2017 and 2019 using the chi-square test for independence with a Yates correction. *P*<.01 was taken as the level for significance.

### Qualitative Data Analysis

Qualitative data from the open-ended questions were captured on the web using Google Forms, exported into an Excel spreadsheet, and subjected to thematic analysis using the 6-step approach to thematic analysis by Braun and Clarke [26]. The qualitative responses were read with the stated question in mind, after which a set of themes was developed by members of the research team (FM and JKM). The responses were reread and assigned to themes, which were further refined as more themes emerged. The responses were reread a third time (JKM), and the themes were reviewed. Discrepancies were presented to a third author (VB) and discussed until consensus was reached.

### Ethical Considerations

The study was approved by the human research ethics committee of the University of Cape Town (HREC 280/2020), CMSA, South African Committee of Medical Deans, and Health Professions Council of South Africa. Participation was voluntary, and informed consent was obtained before inclusion in the study. This study, which involved human participants, was performed in accordance with the Declaration of Helsinki. All examination candidates gave signed informed consent before participation in the study. All methods were conducted in accordance with relevant guidelines and regulations. Declared consent for publication is not applicable as no identifying images or information was used in the paper.

## Results

### Participants

Table 1 shows that during the first 12 months of the COVID-19 pandemic, from June 2020 to June 2021, a total of 3105 candidates registered for postgraduate certification examinations conducted by CMSA. After announcing a transition to web-based examinations, of the 3105 candidates, 351 (11.3%) candidates withdrew (written: 293/351, 83.5%; oral: 58/351, 16.5%); overall participation was 88.7% (2754/3105) during the pandemic. Of the 2754 entrants, 1912 (69.43%) passed the written components of the certification examinations, and 1525 (55.37%) were invited to a web-based SOE, of which 1467 (53.27%) attended the SOE (1467/1525, 96.2% participation).

**Table 1:** Candidates undertaking the web-based certification examinations hosted by the Colleges of Medicine of South Africa^a,b^.

	Total, n (%)	2020, n (%)	2021, n (%)
Candidates who registered for certification examinations	3105 (100)	2005 (64.57)	1100 (35.43)
Candidates who withdrew from the online written examinations	293 (100)	266 (90.78)	27 (9.22)
Candidates who passed the online written examinations	2799 (100)	1732 (61.88)	1067 (38.12)
Candidates who were invited to the web-based SOE^c^^,^^d^	1525 (100)	646 (42.36)	879 (57.64)
Candidates who withdrew from the web-based SOE	58 (100)	24 (41.38)	34 (58.62)
Candidates who participated in the web-based SOE	1467 (100)	622 (42.39)	845 (57.6)
Candidates who passed the web-based SOE	1159 (100)	497 (42.88)	662 (57.12)
Candidates who were admitted (passed the online written and web-based SOEs)	1159 (100)	497 (42.88)	662 (57.12)

^a^Pass rate of virtual SOE: total=79% (1159/1467), year 2020=79.9% (497/622), year 2021=78.3% (662/845).

^b^Overall pass rate: total=86.5% (2433/2812, year 2020=91% (1583/1739), year 2021=79.2% (850/1073).

^c^SOE: structured oral examination.

^d^Only exit-level certification examinations include virtual SOEs.

### Questionnaire Completion

The web-based survey was administered to the first cohort of candidates who participated in web-based SOEs between June 1, 2020, and November 15, 2020. Of the 622 potential participants, 542 (87.1%) completed the web-based survey.

### Are Web-Based SOEs Acceptable to Candidates?

#### Candidates’ Perceptions of Web-Based SOEs

Table 2 shows that, broadly speaking, candidates expressed a positive opinions about web-based SOEs. They considered web-based SOEs to be a fair method of examination (374/542, 69%), which tested their clinical reasoning and insight appropriately (396/542, 73.1%). Furthermore, case scenarios were of adequate complexity (417/542, 76.9%), station length and overall examination length were appropriate (364/542, 67.2%), more cases better displayed their knowledge (363/542, 66.9%), and examination material was well presented (369/542, 68.1%). Less than one-third (173/542, 31.9%) felt that real patient encounters would have been preferred, and a small minority (152/542, 28%) would have preferred an in-person oral examination.

**Table 2:** Candidates’ perceptions of web-based structured oral examinations (SOEs).

Candidates’ perceptions of web-based SOEs		Level of agreement (n=542), n (%)		
		Agree	Neutral	Disagree
**Adequate method of assessment**				
	The exam adequately tested my clinical reasoning, judgement, insight, and decision-making	396 (73.1)	108 (19.9)	38 (7)
	The case scenarios (examination questions) were appropriate to assess an entry-level specialist or subspecialist	417 (76.9)	92 (16.9)	33 (6.1)
	The total length of the examination was appropriate	364 (67.2)	0 (0)	178 (32.8)
	The average time for each station/case was appropriate	364 (67.2)	0 (0)	178 (32.8)
	Having real patients would have improved the quality of the examination	173 (31.9)	168 (30.9)	201 (37.1)
	It would have been preferable to have a local examiner present with me to ask the questions	152 (28)	157 (28.9)	233 (42.9)
**Fair method of assessment**				
	The use of a larger number of case scenarios rather than the historically smaller number of cases gave me a better chance to show my capability	363 (66.9)	141 (26)	38 (7)
	In my opinion, this was a fair examination	374 (69)	130 (23.9)	38 (7)
**Quality of the examination material**				
	The examination material was clearly presented	369 (68.1)	103 (19)	70 (12.9)

#### Candidates’ Perceptions of Zoom for Hosting Web-Based SOEs

The results in Table 3 show that most candidates found the Zoom platform to be acceptable for hosting web-based SOEs (434/542, 80.1%) and considered it to be a fair examination technique (396/542, 73.1%). Most reported that Zoom was technically adequate: 80.1% (434/542) could clearly see and hear examiners, and 69% (374/542) said that video and image quality was adequate. SOEs conducted via Zoom were associated with personal cost-saving (434/542, 80.1%) and time-saving (428/542, 78.9%) benefits because candidates were spared the trouble of traveling. Approximately two-thirds of the participants (336/542, 61.9%) indicated that CMSA examinations should be conducted in the same manner in the future.

**Table 3:** Candidates’ perceptions of the use of Zoom for hosting web-based structured oral examinations (SOEs).

Candidates’ perceptions of the use of Zoom for hosting SOEs		Level of agreement (n=542), n (%)		
		Agree	Neutral	Disagree
**Adequate for hosting web-based** **SOEs**				
	I found it acceptable to have examiners conduct the examination using Zoom	434 (80.1)	81 (14.9)	27 (4.9)
**Fair method of assessment**				
	Conducting oral examinations using Zoom is a fair examination technique	396 (73.1)	108 (19.9)	38 (7)
	The CMSA^a^ should continue to run the exams using Zoom as opposed to a face-to-face process (preferable)	336 (61.9)	125 (23.1)	81 (14.9)
**Audiovisual quality**				
	I could see the examiners clearly on the computer screen	434 (80.1)	59 (10.9)	49 (9)
	I could hear the examiners clearly on the Zoom call	437 (80.6)	73 (13.5)	32 (5.9)
	Images and videos used were of adequate definition or quality to be considered a fair examination	374 (69)	108 (19.9)	60 (11.1)
**Personal benefits**				
	The personal *cost saving* of having the exam locally using Zoom was worth it	434 (80.1)	70 (12.9)	38 (7)
	The *time saved* by being able to participate in the exam locally was worth it	428 (78.9)	65 (11.9)	49 (9)

^a^CMSA: Colleges of Medicine of South Africa.

### What Technical Challenges Did Candidates Encounter While Using Zoom?

The first 2 cycles of virtual SOEs required 6258 Zoom calls, all of which were successfully completed on the appointed examination day. All candidates were able to complete their web-based SOE using Zoom. Altogether, 173 technical challenges were reported during the first examination cycle (2020). All (173/173, 100%) were successfully resolved. Of the 173 challenges, a total of 164 (94.8%) were specifically described in the open-ended section of the survey. Overall, 3 major themes were identified: connectivity-related issues with poor-quality connection, disconnection, and laptop battery failure; poor audio quality of Zoom call with sound delay, poor quality or interruption, and low sound intensity from examiners sitting very far from the microphone; and poor image quality on Zoom call with video or photograph either very small or unclear:

Some issues with connectivity in one station. [It] was quickly resolved and another examiner took over [the] questions so I don’t feel disadvantaged due to it.

Candidate 383

Electricity load-shedding occurred with the generator being switched on at the venue. The volume of the speaker was low and the invigilator increased the volume.

Candidate 263

Sometimes it would appear as though an examiner had frozen on the screen where their broadband signal had become temporarily weak. This did not disturb the call entirely given that we had the buffer 5 minutes in case of technical glitches.

Candidate 128

Some audio disturbance, and can hear the exam next door is a bit distraction.

Candidate 414

### On the Basis of Candidates’ Experiences, How Could We Enhance the SOE Experience?

#### Overview

Thematic analysis of the open-ended questions allowed us to better understand the positive and negative experiences of candidates undertaking web-based SOEs using Zoom. We identified 27 themes, which we categorized into 4 overarching themes, as shown in Table 4.

**Table 4:** Themes from qualitative analysis of candidates’ responses.

Themes	Subthemes	
	Positive experiences	Negative experiences
Examination design and content	No home-ground advantage Standardized scenarios and questions Fair questions Expanded case number Less intimidating Helpful mock examination Less examiner-examiner interaction	No preparation time No station preview Limited media engagement Limited case discussion Long examination waiting times
Examination environment	Helpful and friendly proctoring staff On-site IT support	Variable audiovisual quality Obtaining assistance Lack of earphones Lack of screen share control No hard copies of case scenarios
Examiner-candidate interaction	More examiner interaction without patients	Examiner visibility on screen Virtual examiner engagement Loss of visual cues from examiners
Personal benefits for candidates	Travel convenience Financial saving Home comforts Less interruption	—^a^

^a^No negative personal benefits reported.

#### Examination Design and Content

Candidates endorsed the lack of a *home-ground* advantage:

The fact that all candidates are on the same footing. The home candidates have a clear advantage when it comes to clinical OSCE with patients. Those travelling to other provinces do so at great cost and emotional stress. The examination as it was done now should be the way forward.

Candidate 257

Candidates appreciated the standardization of case scenarios and examination questions:

Excellent standardisation. All candidates had the same examiners, same questions, same experience. Removed all bias from exam.

Candidate 22

A great number of case scenarios also contributed to a fair examination in the virtual context than in previous in-person oral examinations:

I really enjoyed that the number of cases in the examination was increased as I think that one is able to get a good impression of a candidate if they are examined over broader spectrum rather that certain cases as depicted by the examiners, so that if a candidate does badly in one case they still have a shot at redemption.

Candidate 238

It was good to have a wider range of cases including paediatric and emergency cases that we normally would not be examined on. I found the experience of virtual presence of the examiners less intimidating.

Candidate 33

Candidates also felt that virtual oral examinations were fair and more transparent because there was limited examiner-to-examiner interaction:

I think it was also great that examiners where not in the same room and each examiner examined the candidate independently [examiner not aware what another examiner scored for a particular candidate] and that they did not discuss what mark should be given to a particular candidate. Think this brings fairness and transparency.

Candidate 238

Mock examinations were also perceived favorably. However, the long waiting times before and after the examination were negatively received:

The Zoom Mock exam was reassuring, thank you for giving us clarity. Though it was unpleasant to wait for 2 hours before exams start, I did appreciate the session slots. It was very organized, I commend the team.

Candidate 487

Unfortunately the duration of the examination process is tedious. If this platform goes ahead it would be preferable to have wrapped up the exams in a shortest time frame.

Candidate 485

Negative experiences that candidates felt could be improved included an overview of the station before it begins:

Ask examiners to provide a brief overview of the length of the station/number of questions in order to plan answers/timing accordingly.

Candidate 167

Participants also wanted to add preparation time to each station:

Inadequate time to review slides and prep, many stations required you time to grasp the concepts and integrate the information. It was difficult to do in the time frame provided. Difficult to read the slides and process information and feedback [to examiners].

Candidate 63

Candidates wanted better control of the media included in the case scenarios, for example, scrolling through computed tomography imaging studies:

Inability to interact with images and unavailability of dynamic acquisitions.

Candidate 222

Unable to control imaging myself to make an assessment.

Candidate 386

They also wanted the option of viewing video clips more than once:

For the video station, to have an option of watching the video twice if you would like to do so because in a real case scenario when examining a patient, you can repeatedly ask the person to do something. Some of the stations should be made realistic, like the emergency station, in a real-life situation, the patient comes in, you don’t get history first the ask to see the patient.

Candidate 481

Although the candidates enjoyed the breadth of knowledge that was assessed, they wanted an additional way to portray their clinical maturity and insight:

Feeling unable to give a proper account of my knowledge. There is no room for clinical discussion or arguments when answering against a rubric all the time. Maybe one or two stations would be valuable where there’s no rubric and just a discussion around a topic or a case etc.

Candidate 90

Other negative experiences included a reflection of case scenario design and highlighted the importance of designing appropriate questions for a virtual examination environment:

The examination should have either a model or a device with which the candidate could demonstrate their skills [if the candidate is being asked to demonstrate use of such device]. Adequate resolution of images is also a necessity.

Candidate 70

Participants suggested administering the case scenarios in a more authentic way to better reflect a real patient encounter:

Because there are no real patients perhaps show pictures of not only the positive findings that the examiner needs to candidate to comment on, but also important negative clinical findings. For example, if the main pathology is in the external ear, it would be helpful to show a picture of a normal TM [tympanic membrane]. When the candidate now interprets the scan, it helps to know that the middle ear was normal.

Candidate 33

#### Examination Environment

Candidates really appreciated the helpful, compassionate, on-site proctoring staff:

Invigilators were very supportive and accommodating, they gave us 5-star treatment, Thank you very much. Examiners were very empathetic.

Candidate 487

Moreover, they specified that efficient troubleshooting and reliable technical support were important:

Well organized, flowed well with on-the-spot problem solving, excellently managed.

Candidate 421

Having an efficient way to call for rapid technical assistance was identified as a possible improvement:

...Have a buzzer or bell in the exam room to enable you to ask for assistance if signal is lost. Or having a technical person in the room while you are busy.

Candidate 505

Clear instruction about the conduct of the examination was also considered important:

Inform the candidates how the examination will be conducted.

Candidate 443

...Explain the role of the technician prior to examination...

Candidate 44

Familiarity with the virtual platform was highlighted as an important positive finding. Candidates liked that the virtual platform offered an emotionally and professionally neutral venue. They also reported that the virtual examiner-candidate meeting was less anxiety provoking:

I was comfortable having the oral in a Zoom meeting in a neutral venue that is outside the laboratory. I felt less anxiety than for face-to-face interactions...

Candidate 166

The zoom meeting was less stressful, I am generally anxious, I was less anxious.

Candidate 98

Less anxiety as examiners are not present with me.

Candidate 78

Sound quality issues included concerns about struggling to hear examiners:

Not always being able to hear clearly.

Candidate 241

Concerns about not being heard by examiners were also mentioned:

Worrying about not projecting well/being audible.

Candidate 462

Earphones to enhance sound quality were suggested:

Using earphones might assist with sound quality...

Candidate 505

Candidates would have liked paper to write on:

Allow you to have blank paper with you during the question session to better structure your thoughts.

Candidate 349

They also wanted hard copies of the case scenarios during the virtual SOEs:

Provide a paper copy of the case scenario and questions. Increase the time allowed for answering.

Candidate 60

#### Candidate-Examiner Interaction

Examiners’ interaction with candidates was an important theme, and both positive and negative encounters were mentioned by candidates.

Candidates found the virtual encounters to be less intimidating:

I found that having the examiners visible but not face-to-face made the exam less intimidating.

Candidate 182

They also reported that examiners’ engagement with candidates was improved because of the absence of patients:

I felt that the examiners engaged with you more during the zoom meeting because there were no patients.

Candidate 226

However, the virtual environment required more examiner engagement with candidates to reassure them that they were being heard:

Due to lack of in person examiners, examiners need to be engaging and acknowledge the participants response. Not guide or prompt, but make it clear to the participant that they could be heard.

Candidate 123

Furthermore, candidates did not approve of examiners being distracted during virtual examinations conducted in their homes:

Examiners should have their phones off and not be distracted by other factors if examining from home.

Candidate 130

Examiners’ visibility on the screen was viewed both positively and negatively:

I wasn’t actually told I had to have the examiners’ video visible, so I switched it off and found this a much more pleasant experience than what I had been expecting based on colleagues’ related experiences of previous examinations.

Candidate 251

All examiners must be visible during the exam.

Candidate 459

The importance of examiners’ eye contact with the camera was specifically mentioned:

Does lose some of the interpersonal contact. Can be distracting if the examiner doesn’t look at the camera/interact.

Candidate 125

Candidates noted that there was less “positive” examiner cueing because it was not possible to read the examiners’ body language:

Lacking positive reassurance from visual cues given by examiner in a normal setting. Although it is not a given that you would receive that even in a face to face oral.

Candidate 352

Not being able to read body language.

Candidate 362

Less able to elicit nonverbal communication/feedback with mock patient and examiner, but this was not a major factor.

Candidate 427

#### Personal Benefits for Candidates

Candidates found the examination design to be overwhelmingly positive. It was associated with financial savings, reduced levels of anxiety, travel convenience, and availability of home comforts:

No travel. No need to adjust to unknown environment and clinical setting. Costs saved.

Candidate 349

To be based locally in a huge relief. It alleviates a lot of the stress during an already very stressful time.

Candidate 365

I liked that it started later, reduced anxiety of traffic and allowed me to sleep better.

Candidate 431

Home, familiar surroundings, and staff very helpful.

Candidate 373

On the basis of the findings of the thematic analysis, we compiled 10 suggestions that could possibly further improve the web-based SOE experience for candidates. They are listed in Textbox 1.

**Suggestions from candidates for enhancing the web-based structured oral examination (SOE) experience.**

**Suggestions from candidates**

- Provide candidates with an overview of time allocation per station or desk timer in station
- Allocate preparation time before station begins
- Design case scenarios such that strong candidates can demonstrate their clinical maturity
- Provide paper for candidates to make notes during virtual SOEs
- Allow candidates to control media content, including scrolling and screen share capability
- Allow candidates to use earphones or headphones to improve audio quality
- Provide dual screen capabilities when additional examination material needs to be viewed
- Allow candidates the option of not seeing their examiners (examiners have their video off)
- Train examiners regarding virtual engagement techniques: eye contact and verbal reassurance
- Minimize the duration of digital isolation while maintaining examination integrity

### Are Pass Rates of Web-Based and In-Person Certification Examinations Comparable?

Most candidates (1159/1467, 79%) who undertook web-based SOEs were ultimately successful in passing their certification examinations and gained admission to the respective member colleges of CMSA. The pass rates for 2020 and 2021 were 91.4% (1583/1732) and 79.7% (850/1067), respectively. The average pass rate for postgraduate in-person certification examinations conducted by CMSA 3 years before the pandemic (2017-2019) was 58.23% (4030/6921). This was significantly lower than the pass rate for the first 2 cycles that included web-based certification examinations (2020: N=8635; *χ*^2^_1_=667; *P*<.001; 2021: N=7988; *χ*^2^_1_=178; *P*<.001).

## Discussion

### Principal Findings

This paper reports about >1500 candidates’ experiences of web-based SOEs conducted via Zoom as part of postgraduate specialty and subspecialty certification examinations hosted by CMSA during the early part of the COVID-19 pandemic. Despite the short time frame in which this transition to web-based examinations was executed, the initiative was well received by candidates. Overall, they perceived web-based SOEs to be a fair and appropriate assessment method, were generally satisfied with the use of Zoom for hosting the examination proceedings, and reported surprisingly few technical challenges. The qualitative data provided a rich analysis of their positive and negative experiences and provided constructive suggestions that could further improve the web-based SOE experience.

An important finding of our study was the observation that candidates undertaking web-based certification examinations performed better than candidates previously undertaking in-person certification examinations. This study did not set out to explore the possible reasons for this observation, but some qualitative observations from candidates may be of interest in this regard. First, candidates reported feeling less intimated by examiners during web-based oral examinations. Second, improved standardization of the examination process using identical case scenarios and standardized questions with marking memoranda may have contributed to fair examination conditions supporting better overall candidate performance. Third, candidates found the overall web-based examination experience to be less stressful, and this may have positively influenced their performance. Fourth, examiners were required to score candidates’ performance independently without conferring before awarding a final score. This limited examiner-examiner interaction may have limited examiner coercion and had a positive impact on the final scores awarded.

### Important Improvement Considerations

On the basis of our qualitative analysis of candidates’ experiences, we formulated 10 suggestions that could further enhance the web-based SOE experience for candidates. These suggestions may be helpful to others currently seeking to improve web-based SOE experiences for candidates. Clear, concise information should be delivered to the candidates ahead of the examination, informing them about the conduct of the examination and approaches to troubleshooting and dealing with technical problems when encountered, especially to ensure that technical issues do not have an impact on examination time. Specific preparation time should be allocated before each case scenario conversation commences. Candidates should have paper in each station to make brief notes during web-based SOEs, if needed. Case design and content should be carefully reviewed to ensure that it is appropriate for a web-based examination, for example, avoid demonstrating the use of equipment that is not present in the web-based examination setting. The *screen share* option on Zoom should be available to the candidate to review images and videos independently. Examiners should be briefed about the importance of visual and verbal engagement with candidates to ensure a more authentic experience for candidates.

### Findings of Other Studies

There are several prepandemic papers reporting favorably about the use of in-person SOEs in medical education [14-21]. Although these reports were encouraging and supported the use of SOEs for assessment, they did not speak in the context of web-based oral examinations for high-stakes postgraduate certification purposes, which is the topic of this paper. When looking specifically at this context, it is apparent that this is an emerging field that has gained momentum during the pandemic. Currently, there are a few recent reports directly relevant to the key findings of this study. Before the pandemic, McGrath et al [27] reported a study comparing web-based oral examinations with in-person oral examinations for anesthesiology residents. The study randomized 35 residents to testing conditions using an immersive learning environment. Although an immersive learning environment is not the same as a web-based oral examination conducted via Zoom, the paper broadly contributes to the conversation about moving postgraduate oral examinations into a web-based setting. It is worth noting that the authors reported similar academic performance in both groups, which is consistent with our findings of the noninferior performance of candidates undertaking web-based oral examinations. In the study, as also seen in our study, most examination candidates preferred the web-based experience, found it to be less intimidating than in-personal oral examinations, and commented about the cost saved by not traveling to testing sites.

Recently, Chaurasia et al [5] conducted a small pilot study of 8 radiation oncology postgraduate trainees and 8 examiners testing the use of web-based oral examinations conducted via Zoom. Candidates were engaged in 8 stations of 25 minutes each, using *breakout rooms*. Similar to us, they found Zoom to be easy to use, adequate for examination purposes, and free of serious technical difficulties and described it as a fairly seamless experience. They used *screen share* and *annotate* for anatomy review, contouring, and treatment plan evaluation for virtual radiation oncology cases. Users described the web-based experiences easier or the same for ease of understanding of the cases and reported preparation to be the same or less time consuming than for in-person oral exams. The authors concluded that a move to web-based oral examinations for postgraduate certification examinations in radiation oncology should be considered as a feasible alternative.

In 2021, a total of 44 senior vascular surgery postgraduate trainees from 17 US training institutions undertook web-based mock oral examinations with 2 remote examiners via Zoom [28]. For each candidate, examiners selected 4 cases from a book of 30 vascular scenarios, and candidate performance was assessed using a standardized scoring sheet. Consistent with our findings, the authors reported no difference in how well the knowledge base of candidates could be examined compared with in-person oral examinations. They also reported that candidates could adequately express their confidence in the web-based setting and concluded that web-based oral examinations are a viable option to consider for examination purposes. They also mentioned the cost saved by remote examinations. The most important considerations highlighted in that paper was the fairness of test grading achieved by using a standardized marking sheet and the equity of test questions achieved by using a standardized set of case scenarios. This speaks to factors that may contribute to favorable candidate performance as already mentioned by us.

In 2022, the Vascular Surgery Board reported about the successful implementation of web-based certification examinations [29]. They reported about the findings of 356 successful candidates who each undertook three 30-minute virtual oral examinations hosted via Zoom. Similar to the findings of our study, the examination process was well received by candidates and found to be technically adequate. Similar to our candidates, they raised concerns about image quality and time constraints to answer all questions but expressed appreciation for good planning and execution, convenience of local examination conditions, and avoidance of travel costs. Overall, candidates were significantly more in favor of continuing web-based examinations as compared with examiners (87% vs 32%; *P*<.001). The authors make a case for the feasibility and convenience of continuing web-based certification examinations beyond the COVID-19 pandemic. Questions about IT support costs and the cost of remote proctoring are also raised in the paper.

The American Board of Obstetrics and Gynecology also recently published the outcome of their web-based oral certification examinations that were also conducted via Zoom [30]. Between 2021 and 2022, a total of 1491 specialty and 830 subspecialty candidates undertook three 1-hour oral examinations using 3 pairs of examiners per candidate. They found that candidates performed similarly in the web-based certification examinations as compared with previous in-person oral certification examinations. Similar to our findings, they experienced few technical difficulties, and candidate satisfaction with the remote examination was high; however, they expressed anxiety about the use of technology (remotely at home or elsewhere). Despite the success of these remote oral examinations, the issues of remote proctoring and the technical burden placed on candidates being examined in their homes have resulted in a decision to return to testing center–based in-person examinations.

The American Board of Surgery also recently published the findings of their first web-based certification examination for general surgery candidates conducted in 2021 [31]. They report about 306 successful candidates who also completed a web-based satisfaction survey after the examination. They also found that the pass rates for web-based oral examinations were no different from those of in-person oral examinations and that the new examination format was well received by candidates. Audio and video quality were adequate, and 78% of the survey respondents indicated that web-based certification examinations were preferred. The authors argue that the findings support the expanded use of this method of assessment.

The American Board of Ophthalmology has also reported about their success in conducting web-based oral examinations via Zoom for >1000 candidates over the past 12 months. Similar to our study, they took the opportunity to improve the standardization of the examination process and used 2 independent examiners. They described the inordinate preparation and planning required to effect a rapid transition from in-person to web-based certification examinations. They also found that the examination was very well received by both candidates and examiners who felt that the ability to assess knowledge, insight, and judgment using web-based oral examinations was the same as in in-person examinations. Positive opinions regarding the use of the assessment method after the pandemic were expressed by a range of stakeholders. However, they did express some ambivalence over the continued use of web-based oral examinations.

### Strengths and Limitations

Most current reports in the literature focus on small numbers of candidates. At the time of writing, this was one of the largest published studies reporting about web-based SOEs for postgraduate certification purposes. There are, however, important limitations in this study. We only reported about the initial 12 months of implementation, and a follow-up report is needed. Candidates may have viewed web-based oral examinations more favorably during the pandemic, and this opinion needs to be reviewed. The most significant limitation of the study is that the opinion of examiners was not captured. This was not possible during the pandemic because examiners were overburdened with additional clinical responsibilities and were setting up a new examination method with very little preparation time before the web-based examinations process began. Clearly, their opinion is needed, and after 36 months of implementation, it will contain a wealth of important considerations. Finally, this is a single-country study, and reports from elsewhere are needed.

### Future Directions

Internationally, the move from in-person to web-based oral examinations, as part of postgraduate specialty certification examination processes, has gathered momentum [2,3,29-32]. There are, however, lively debates about the ongoing use of web-based oral examinations in the postpandemic era, and it seems that opinions are currently divided on the matter [29-32]. Cost and time savings and convenience of arranging and attending are significant factors in favor of remote oral examinations. Major disadvantages of web-based oral examinations include the cost of IT support and remote proctoring [33] and the inability of examiners to assess bedside skills (history taking and physical examination). Increased uptake of workplace-based assessment in postgraduate training programs should effectively address the ongoing concerns about clinical competence. More cost-efficient delivery of IT support and the challenges of remote proctoring require further studies.

### Conclusions

This study highlights the value of conducting formative research about the use of web-based SOEs as part of postgraduate certification processes. We found that this method of assessment was well received by examination candidates, could be conducted via Zoom with surprisingly few technical challenges, and did not have a negative impact on candidates’ academic performance. Our encouraging findings are consistent with early reports from elsewhere that provide positive preliminary evidence supporting the ongoing use of this novel web-based assessment method in postgraduate medical education.

## Appendix

Multimedia Appendix 1 Survey.

## Abbreviations

CMSA: Colleges of Medicine of South Africa
SOE: structured oral examination
