# Supplementary material for: Feasibility and Acceptability of Web-Based Structured Oral Examinations for Postgraduate Certification: Mixed Methods Preliminary Evaluation
Source: JMIR Form Res. 2024 Mar 6;8:e40868. doi: 10.2196/40868 (PMC10919348; doi:10.2196/40868)
Supplement: Multimedia Appendix 1 [file formative_v8i1e40868_app1.docx]

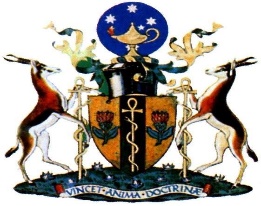


**The Colleges of Medicine of South Africa (CMSA)**

**EVALUATION OF THE REMOTE ZOOM-BASED STRUCTURED ORAL EXAMINATIONS NECESSITATED BY THE COVID-19 PANDEMIC**

Dear Fellowship or Sub-Specialist Certificate Candidate,

To conduct your Fellowship / Certificate examination in the face of COVID-19, The Colleges of Medicine of South Africa (CMSA) has introduced two innovations.

1. The first is to replace the traditional clinical exam with a combination of an abridged Workplace-Based Assessment (Assessment of Competence), a Structured Oral Examination (SOE) and an online short-answer written assessment, where appropriate.
2. The second is to conduct the oral examination by Zoom instead of face-to-face.

The purpose of this survey is to learn about how these two methods fared.

**Ethics approval** has been obtained from the Human Research Ethics Committee of the University of Cape Town.

- By taking this anonymous survey, you agree that your participation is voluntary.
- Your decision to agree or not to participate will have no bearing on your examination results as your name, examination number and contact details are not requested.
- You accept that that any personally identifying data, data collection and analysis of the data will be anonymized such that it will not reveal your identity or be identifiable to you.

If you agree to participate in this survey, please click “Yes” to proceed to the questionnaire.

It should take you no longer than 15 minutes to complete the survey.

**EVALUATION OF THE REMOTE ZOOM-BASED STRUCTURED ORAL EXAMINATIONS NECESSITATED BY THE COVID-19 PANDEMIC**

**PLEASE TICK ON YOUR ANSWER USING THE SPACE PROVIDED**

1. Tick on the exam you sit for

| 1. FCA (SA) Part II  | 19. FC Paed Surg (SA) Final | 35. Dip Allerg (SA)  |
| --- | --- | --- |
| 2. FCD (SA) Orthod Part II  | 20. FC Path (SA) Anat Part II | 36. DA(SA)  |
| 3. FCD (SA) OMP Part II  | 21. FC Path (SA) Chem Part II | 37. Dip For Med (SA) Clin/PathDCH(SA) |
| 4. FC Cardio (SA) Final | 22. FC Path (SA) Haem Part II | 38. Dip For Med (SA) Path |
| 5. FC Derm (SA) Part II | 22. FC Path (SA) Haem Part II | 39. Dip Obst (SA)  |
| 6. FCEM (SA) Part II | 23. FC Path (SA) Micro | 40. Dip Ophth (SA)  |
| 7. FCFP (SA) Final Part A | 24. FC Path (SA) Viro | 41. Cert Cardiology (SA) Phys |
| 8. FC For Path (SA) Part II | 25. FCP(SA) Part  | 42. Cert Cardiology (SA) Paed |
| 9. FCMG (SA) Part II  | 26. FC Plast Surg (SA) Final | 43. Cert Clin Haematology (SA) Path/Phys |
| 10. FCMFOS (SA) Final | 27. FC Psych (SA) Part II | 44. Cert Critical Care (SA) Paed |
| 11. FC Neurol (SA) Part II | 28. FCPHM(SA)  | 45. Cert Critical Care (SA) Anaes, Emerg Med, O&G, Phys,  |
| 12. FC Neurosurg (SA) Final | 29. FC Rad Diag (SA) Part II | 46. Cert Dev Paed (SA)  |
| 13. FCNP(SA) Part II | 30. FC Rad Onc(SA) Part II | 47. Cert Gastroenterology (SA) Paed |
| 14. FCOG(SA) Part II  | 31. FCS(SA) Final | 48. Cert Gastroenterology (SA) Phys |
| 15. FC Ophth (SA) Final  | 32. FC Urol (SA) Final | 49. Cert Gastroenterology (SA) Surg |
| 16. FC Orth (SA) Final | 33. FCMFOS (SA) Intermediate | 50. Cert Gynaecological Oncology (SA)  |
| 17. FCORL(SA) Final | 34. FC Ophth (SA) Intermediate 1B  | 51. Cert ID(SA) Paed |
| 18. FC Paed (SA) Part II  |  |  |
|  |  |  |
|  |  |  |
|  |  |  |

| 52. Cert ID(SA) Phys |
| --- |
| 53. Cert Maternal and Fetal Medicine (SA)  |
| 54. Cert Medical Oncology (SA) Paed |
| 55. Cert Neonatology (SA)  |
| 56. Cert Paediatric Neurology (SA)  |
| 57. Cert Pulmonology (SA) Paed |
| 58. Cert Pulmonology (SA) Phys |
| 59. Cert Reproductive Medicine (SA)  |
| 60. Cert Rheumatology (SA) Phys |
| 61. Cert Trauma Surgery (SA)  |
| 62. Cert Vascular Surgery (SA) |
|  |
|  |
|  |

2. LOCATION/VENUE OF YOUR EXAMINATION

1. Bloemfontein 

2. Cape Town 

3. Durban 

4. East London 

5. Johannesburg 

6. Polokwane 

7. Port Elizabeth 

8. Umtata 

**A. STRUCTURED ORAL EXAMINATIONS**

3. The exam adequately tested my clinical reasoning, judgement, insight, and decision-making.

1 Completely agree

2 Agree

3 Neutral

4 Disagree

5 Completely disagree

4. The case scenarios (examination questions) were appropriate to assess an entry-level specialist /– subspecialist.

1 Completely agree

2 Agree

3 Neutral

4 Disagree

5 Completely disagree

5. The total length of the examination was:

1 Much too long

2 Too long

3 Appropriate

4 Too short

5 Much too short

6. The average time for each station/case was

1 Much too long

2 Too long

3 Appropriate

4 Too short

5 Much too short

7. The examination material was clearly presented.

1 Completely agree

2 Agree

3 Neutral

4 Disagree

5 Completely disagree

8. The use of a larger number of case scenarios rather than the historically smaller number of cases gave me a better chance to show my capability.

1 Completely agree

2 Agree

3 Neutral

4 Disagree

5 Completely disagree

6 The number of cases unchanged from previous examinations

9. Having real patients would have improved the quality of the examination.

1 Completely agree

2 Agree

3 Neutral

4 Disagree

5 Completely disagree

10. Did you experience any technical problems with the way the examination material was presented?

1 Yes

2 No

11. If YES: please provide details

--------------------------------------------------------------------------------------------------------------------------------------------------------------------------------------------------------------------------------

12. In my opinion, this was a fair examination.

1 Completely agree

2 Agree

3 Neutral

4 Disagree

5 Completely disagree

**B.** **EXAMINATION BY ZOOM MEETING**

13. I found it acceptable to have examiners conduct the examination using Zoom.

1 Completely agree

2 Agree

3 Neutral

4 Disagree

5 Completely disagree

14. It would have been preferable to have a local examiner present with me to ask the questions.

1 Completely agree

2 Agree

3 Neutral

4 Disagree

5 Completely disagree

15. I could see the examiners clearly on the computer screen.

1 Completely agree

2 Agree

3 Neutral

4 Disagree

5 Completely disagree

16. Examiners and moderators were visible to you on the screen. Would you have preferred to only have the examiners voice only and no visual of the examiners?

1 Yes

2 No

 17. I could hear the examiners clearly on the Zoom call.

1 Completely agree

2 Agree

3 Neutral

4 Disagree

5 Completely disagree

18. Images and videos used were of adequate definition/quality for the process to be considered a fair examination.

1 Completely agree

2 Agree

3 Neutral

4 Disagree

5 Completely disagree

6 None used

19. The personal “cost saving” to me of having the exam locally using Zoom was worth it.

1 Completely agree

2 Agree

3 Neutral

4 Disagree

5 Completely disagree

20. The “time saved” by being able to participate in the exam locally using Zoom was worth it.

1 Completely agree

2 Agree

3 Neutral

4 Disagree

5 Completely disagree

21. Conducting oral examinations using Zoom is a fair examination technique.

1 Completely agree

2 Agree

3 Neutral

4 Disagree

5 Completely disagree

22. The CMSA should continue to run the exams using Zoom as opposed to to a face to face process.

1 Completely agree

2 Agree

3 Neutral

4 Disagree

5 Completely disagree

23. Did you experience any technical problems during the Zoom examination?

1 Yes

2 No

24. If YES: please provide details and explain how it was resolved/managed.

--------------------------------------------------------------------------------------------------------------------------------------------------------------------------------------------------------------------------------25. How could the CMSA improve examinations conducted using Zoom?

--------------------------------------------------------------------------------------------------------------------------------------------------------------------------------------------------------------------------------

26. What were the positive experiences for you as a candidate in this examination?

--------------------------------------------------------------------------------------------------------------------------------------------------------------------------------------------------------------------------------

27. What were the most negative experiences for you as a candidate in this examination?

--------------------------------------------------------------------------------------------------------------------------------------------------------------------------------------------------------------------------------

28. Would you like to provide any other feedback that may assist the CMSA with regards to conducting Structured Oral Examinations using Zoom?

**We thank you for your participation**
